# Artificial Intelligence Teaching as Part of Medical Education: Qualitative Analysis of Expert Interviews

**DOI:** 10.2196/46428

**Published:** 2023-04-24

**Authors:** Lukas Weidener, Michael Fischer

**Affiliations:** 1 Research Unit for Quality and Ethics in Health Care UMIT TIROL – Private University for Health Sciences and Health Technology Hall in Tirol Austria

**Keywords:** AI technology, artificial intelligence, clinical context, expert interviews, health care, medical curriculum, medical education, medical school, medical student, medicine

## Abstract

**Background:**

The use of artificial intelligence (AI) in medicine is expected to increase significantly in the upcoming years. Advancements in AI technology have the potential to revolutionize health care, from aiding in the diagnosis of certain diseases to helping with treatment decisions. Current literature suggests the integration of the subject of AI in medicine as part of the medical curriculum to prepare medical students for the opportunities and challenges related to the use of the technology within the clinical context.

**Objective:**

We aimed to explore the relevant knowledge and understanding of the subject of AI in medicine and specify curricula teaching content within medical education.

**Methods:**

For this research, we conducted 12 guideline-based expert interviews. Experts were defined as individuals who have been engaged in full-time academic research, development, or teaching in the field of AI in medicine for at least 5 years. As part of the data analysis, we recorded, transcribed, and analyzed the interviews using qualitative content analysis. We used the software QCAmap and inductive category formation to analyze the data.

**Results:**

The qualitative content analysis led to the formation of three main categories (“Knowledge,” “Interpretation,” and “Application”) with a total of 9 associated subcategories. The experts interviewed cited knowledge and an understanding of the fundamentals of AI, statistics, ethics, and privacy and regulation as necessary basic knowledge that should be part of medical education. The analysis also showed that medical students need to be able to interpret as well as critically reflect on the results provided by AI, taking into account the associated risks and data basis. To enable the application of AI in medicine, medical education should promote the acquisition of practical skills, including the need for basic technological skills, as well as the development of confidence in the technology and one’s related competencies.

**Conclusions:**

The analyzed expert interviews’ results suggest that medical curricula should include the topic of AI in medicine to develop the knowledge, understanding, and confidence needed to use AI in the clinical context. The results further imply an imminent need for standardization of the definition of AI as the foundation to identify, define, and teach respective content on AI within medical curricula.

## Introduction

### Background

Artificial intelligence (AI) has been of broad scientific interest in medicine for over a decade. This is reflected in the publication of more than 18,000 scientific publications mentioning AI-related terms in that time. AI is expected to revolutionize health care systems around the world. Apart from the economic benefits, AI is expected to make health care more efficient for both patients and health care professionals [1]. Improvements are expected to reduce clinician’s workload and leave more time for patient–practitioner interaction [1,2].

With increased public and scientific interest, research into the potential challenges of AI is becoming more commonplace. Recent developments in the use and handling of algorithms in AI applications have raised highly relevant ethical concerns that need to be addressed, in addition to crucial questions regarding patient safety and data [3]. These include questions regarding potentially biased decision-making, the liability in case of any mistakes, and effects on the physician–patient relationship [4].

Researchers propose that addressing potential challenges regarding the use of AI in medicine requires adequate knowledge of the technology [5,6]. Furthermore, studies have shown that early acquisition of knowledge and competencies can increase the acceptability of new technology like AI [7,8]. Recent publications suggest that since medical education is considered to be the basis of the medical profession, integration of AI into the curriculum must occur early and comprehensively [9].

To prepare future generations of physicians for the use of AI within the rapidly changing health care system, education needs to adapt to the new challenges. As the development of new curricula modules and teaching content is a time-intensive and complicated process due to traditional structures and accreditation procedures, significant research is needed to define relevant competencies and teaching content regarding AI in medicine.

### Defining AI

AI has been a topic of interest in computer science since the 1950s [10]. However, due to the often-prevailing heterogeneity in the definition of AI on the part of science and the public, it is essential to present the definition of AI on which this publication is based. This will facilitate not only the interpretation of the following results but also the discussion that follows.

A distinction can be made between so-called strong AI and weak AI. “Strong AI” defines an AI whose intellectual abilities are comparable to those of humans [11]. However, a uniform definition of AI is hampered by the lack of a uniform definition of intelligence as such, which also affects the feasibility of “strong AI” [12]. The term “weak AI” is used to define an AI that is capable of performing certain tasks that may be comparable to humans due to its selective and specific “intelligence” [13]. The “weak AI” can be further divided into the so-called symbolic AI and statistical AI [13]. While “symbolic AI” is based on rules or instructions predefined by humans for the execution of a certain task, “statistical AI” aims to establish correlations that can be established from patterns in the analyzed data itself.

The application areas of “symbolic AI” in medicine mainly include rule-based expert systems, where the rules to be followed by the AI have been previously defined by experts. Clinical decision support systems can be used in patient care, for example, to support doctors in diagnosis and treatment [14]. The subfield of “statistical AI” also includes so-called machine learning (ML), which is the focus of scientific research, especially in the field of medicine. The core of ML is the ability to learn from data without being explicitly programmed to do so. ML also includes the subarea of so-called deep learning, in which artificial neural networks are used to develop information processing similar to that of the human brain [13]. Current application areas of ML in medicine include, for example, the analysis of image-based data in terms of detecting skin cancer or suspicious lesions in mammograms [1,15]. Although there is research interest in developing applications based on “strong AI” to be used in the field of medicine, there are currently no established use cases [16].

The present publication is based on the definition of “weak AI” with its subdomains and all results should be interpreted against this background.

### Objective

The study was conducted to explore essential knowledge and understanding regarding AI in medicine, relevant to define curricula teaching content within medical education. The results should provide the foundation for the improvement of the education of medical students and the medical curriculum.

## Methods

The following section of this study aims to provide a detailed description of the study design, data collection, and data analysis techniques used in this research. The methods used in this study were chosen to ensure the validity and reliability of the results and to ensure that ethical standards were met.

### Study Setting

The study, conducted from September to November 2022, aimed to identify relevant knowledge and understanding of AI-related teaching content in medical education using semistructured expert interviews. From the total of 68 initially identified and contacted experts in the field of AI in medicine and health care (including information technology, medical informatics, and medicine), we were able to include 12 in this study. Most experts were based in Germany (n=10), with 2 experts being included from Austria. For the qualitative data collection, we defined experts as individuals who have been engaged in full-time academic research, development, or teaching in the field of AI in medicine for at least 5 years.

Experts were recruited by email and personal recommendation by the participants. Of the total of 12 included experts, half were primarily working in the field of research and practical development of AI-based applications in the field of medicine (eg, a researcher at the German Research Centre for Artificial Intelligence). The remaining 6 experts were primarily associated with teaching and research in the field of medical informatics, AI, and digital medicine as part of the medical curriculum (eg, professor for medical informatics). As the experts were primarily recruited by email, an email address that was not publicly accessible through a web-based search was an exclusion criterion.

Additional exclusion criteria were no or less than 5 years of experience in the field of AI in medicine, a lack of consent to the transcription or voice recording as well as a missing current or recent involvement in projects related to the research, development, or teaching of AI in medicine.

### Ethics Approval

The Research Committee for Scientific Ethical Questions (RCSEQ) of the UMIT TIROL – Private University for Health Sciences and Health Technology, Hall in Tirol, Austria, granted ethical approval for the study.

### Data Collection

Web-based interviews were conducted, using the Cisco Webex Meeting application. The meetings were recorded using an analogous voice recorder. We obtained consent from the participants before conducting the interviews, including their agreement to be recorded and their data to be used for research purposes. As part of the interview, a semistructured guideline was used. The guideline included questions about the experts’ education and experience in AI, the anticipated impact of AI in medicine, as well as key competencies required for use of AI in medicine, and possible teaching content (please see the supplementary information for the interview guideline). On average, the interviews lasted for 35 minutes.

### Data Analysis

The recorded interviews were transcribed manually with the help of the transcription software f4transkript and a transcription service provider was used to transcribe some of the transcripts. Transcription followed the established rules of Dresing and Pehl [17]. To analyze the transcripts, qualitative content analysis by Mayring with inductive category formation was used with the help of the software QCAmap (version 1.2.0) and Microsoft Excel (version 16.66) [18]. The data were coded and categorized based on themes related to the objective of this study.

## Results

As a result of the qualitative content analysis, we defined 3 main categories (“Knowledge,” “Interpretation,” and “Application”) with a total of 9 subcategories. Each of the subcategories is defined by quotes from the participants to highlight the procedure and the original meaning. An overview of the 3 main categories with all associated subcategories is shown in Table 1.

**Table 1:** Overview of the 3 defined main categories with the associated 9 subcategories.

Main categories	Subcategories
Knowledge	Basic understanding of artificial intelligence Statistics Ethics Data protection and regulation
Interpretation	Critical reflection Associated risks Data basis
Application	Practical skills Trust

### First Main Category: “Knowledge”

Based on the results of the qualitative content analysis, the first main category was defined. Given the interdisciplinary data collection, the “knowledge” main category summarizes suggested knowledge, which medical students should learn regarding the topic of AI in medicine as part of their education.

#### Subcategory 1: “Basic Understanding of AI”

The first subcategory “basic understanding of AI” highlights the need for basic knowledge and definitions, without an in-depth understanding:

But that's not, in my opinion, about people really understanding the technology down to the smallest detail and being able to implement and train on things themselves. I don't think they need that.

Interview 7

#### Subcategory 2: “Statistics”

The second subcategory “statistics” relates to the good statistical knowledge needed to understand AI, which was mentioned by half of the experts.

The basis is statistics. (...). So that's the basis, because these learning AI methods are all based on statistics.

Interview 5

This subcategory should also account for the importance of understanding probabilities and their application within medicine. Especially with AI-based applications, statistical knowledge will play a key role in the interpretation of results, which will be further addressed in the second main category.

#### Subcategory 3: “Ethics”

Half of the interviewed experts mentioned the need for an understanding of ethical competencies related to the use of AI in medicine, which is captured in the third subcategory “ethics.”

And then just ethical competencies and I think that has a high requirement (...)

Interview 10

The use of AI-based applications in medicine requires adequate ethical competencies to address the new challenges arising through the interaction with patients and the usage of their data. This does not only refer to the well-known “black-box” phenomena of deep learning or potential bias through unrepresentative training data but rather addresses the topics like the medical self-imagine or the physician–patient relationship too. Although ethics has a long tradition within medical curricula, it also needs to adapt to new technological developments in medicine to address associated challenges and discussions.

#### Subcategory 4: “Data Protection and Regulation”

The last subcategory “data protection and regulation” of the first main category summarizes the need for an understanding of data protection laws and regulations concerning the use of AI in the clinical context, mentioned by 4 of the interviewed experts.

(...) where we have to have a good idea of how we can use it, but also what the legal limitations of the whole thing are.

Interview 10

The need for an understanding of data protection laws does not only apply to the use of AI in medicine but is of increasing significance due to the accelerated digitalization of medicine. An understanding of the regulation regarding the use of AI in medicine can help to prevent uncertainties and potential disapproval by users.

### Second Main Category: “Interpretation”

The second main category “interpretation” accounts for the high importance to interpret and evaluate the results provided by AI-based applications in medicine. This main category summarizes the statements related to the evaluation of results and should highlight the importance of sufficient knowledge and competencies needed to address all associated challenges.

#### Subcategory 1: “Critical Reflection”

The first subcategory “critical reflection” addresses the need for adequate knowledge and understanding to question the results yielded from AI-based applications critically.

(...) also of the possibilities to critically question these things.

Interview 4

The ability to critically reflect and question the results shows the importance of adequate teaching of content relating to AI in medicine. As with any traditional technology or application, AI-based applications are not free of mistakes, which in the clinical context can have significant consequences.

#### Subcategory 2: “Associated Risks”

As users need to be aware of potential consequences and risks associated with the results provided by AI, the second subcategory “associated risks” reflects the answers of 5 of interviewed experts:

(...) also what are the, yes, risks? What can go wrong? Well, the AI also makes mistakes, of course.

Interview 2

One of the most mentioned risks was related to false-positive results provided by AI. Without any critical questioning of the results, this can lead to unnecessary treatments for the patients. Although this might be of minor significance in the case of additional physical examination, it could lead to additional exposure to radiation or punctations. Although false-positive results can lead to more imminent negative consequences, the mentioned consequences of false-negative results can be of major significance too in case a disease is not recognized and treated. False-negative or positive results highlight the need to be aware of the associated risks related to the results of AI-based applications in medicine. Furthermore, critical reflection of the results is not only connected to potential associated risks, but rather to an understanding of the data that were used to train AI applications.

#### Subcategory 3: “Data Basis”

The third subcategory of the second main category “data basis” represents the statements of 4 of the experts and describes the need for a good understanding and reflection of the data used in the development process of the AI-based application.

And, of course, you also have to think about the data that might be fed into it now, do they make sense? Are they representative?

Interview 2

Both are important requirements to interpret the results and are closely associated not only with the other subcategories of this main category but rather with the subcategories from the first main category too. Without a basic understanding of statistics and how AI-based applications work, it is hard to understand the need for representative data samples. Potential bias makes ethical competencies necessary to interpret and critically question the results based on the data basis. This subcategory does not only refer to the need for an understanding of whether the data basis is representative of the current patient, but rather the imminent need to understand that current AI applications have very narrow use cases. To prevent false diagnosis and associated consequences, it is necessary to critically reflect on the unreliable results that can arise from deviation from the specific use case.

### Third Main Category: “Application”

Analysis of the interviews yielded a third main category named “application.” This category comprises 2 subcategories and summarizes the requirements to apply AI-based applications in clinical practice.

#### Subcategory 1: “Practical Skills”

The first subcategory “practical skills” addresses the practical skills required, to use AI-based applications of any kind.

In clinical practice, the most important thing is actually the practical application.

Interview 1

This subcategory further includes basic technological understanding and skills needed, to apply any software application. Based on the feedback from half of the interviewed experts, this includes for example competency to use hardware like desktop computers, including keyboard and mouse or operating software used in the clinical context. Moreover, this subcategory summarizes the knowledge and understanding needed to apply AI software within the clinical workflow. Users need to understand whether it makes sense to use the applications and how they can be used to improve the workflow in clinical practice.

#### Subcategory 2: “Trust”

The second subcategory “trust” represents a base layer needed to use any technology. This subcategory relies on adequate knowledge (first main category) and teaching within the medical curricula. The absence of teaching as part of the medical curriculum could further lead not only to the lack of trust and potentially the disapproval of the application, but could also lead to a blind trust, which can have significant consequences as part of the interpretation of results.

Creating trust, but not blind trust.

Interview 12

Creating trust not only concerning the use of AI-based applications but rather trust regarding the own competencies in the process of applying AI-based applications within the clinical context is one of the challenges that can be addressed as part of medical education.

## Discussion

### Principal Findings

The results indicate the significance of the integration of teaching content regarding AI as part of the medical curriculum. All experts interviewed agreed on the importance of teaching AI content in the medical curriculum, which echoes the current state of literature [6,8,19]. Although an interdisciplinary approach to data collection was chosen, there was significant agreement on the relevant knowledge and competencies required to use AI in the clinical context.

This agreement is reflected through the definition of the 3 main categories (“Knowledge,” “Interpretation,” and “Application”). Most experts recommended that medical students should only receive basic knowledge of current AI models and terminology, as they will not be required to develop or train AI-based applications themselves, which is also in line with recommendations of current publications [6,20]. However, the experts disagreed about the definition of the knowledge that medical students should acquire as part of medical education. For example, some experts were convinced that the responsibility of ensuring the ethical and unbiased development of AI-based applications falls on developers and companies, rather than on medical students, and therefore the need for teaching ethical aspects of AI in medicine is considered to be low. Current publications suggest that even though developers of AI-based applications should do their best to consider ethics during the whole development process, users must be aware of potential ethical issues and challenges arising through the use of AI in medicine [21-23].

The practical challenges and barriers of implementing new teaching content, such as the need for the renewal of accreditation or sufficient knowledge of the teaching staff, further reinforce the recommendations of the experts to only facilitate a basic level of knowledge acquisition of AI as part of the medical education [24]. The experts interviewed for this study agree on the need for opportunities to specialize in AI based on the student’s interest and the requirement for ongoing training programs and extracurricular activities suggested by current publications [7,20,25]. The transfer of knowledge on the topic of AI in medicine is required to build an understanding and competencies needed to interpret the results provided by an AI-based application and apply the new technology within the clinical context.

For many of the interviewed experts, the ability to interpret results provided by AI applications concerning the data basis and the associated risks is highly important when it comes to preferred teaching outcomes. The results from this study confirm the imminent need for an early and conscientious implementation of curricula teaching content on AI, as suggested by earlier studies [9,26,27]. For example, a study published in 2021 found that >90% of medical students anticipate new social and ethical challenges related to the use of AI in medicine [28]. Moreover, current publications on the knowledge and perception of medical students concerning AI show that the overall level of confidence and knowledge is comparably low, given the anticipated impact in the field of medicine [28-30].

### Lack of Standardization

The experts’ statements reveal a disagreement and lack of standardization in the definition of AI. Recent publications on the integration and teaching of AI within medical education commonly lack a specific and dedicated definition of AI [6,8,19]. Given that the definition of AI should be considered the necessary foundation to identify, define, and teach respective content on AI within medical curricula, the lack of standardization has further limited the comparability of current scientific publications significantly. For example, the demanded awareness of potential limitations, risks, and opportunities within the scientific literature and the experts’ statements of this study may vary depending on whether applications based on statistical or symbolic AI are considered [6,19].

The need for standardization in the definition of AI as a foundation for related teaching content is further emphasized by the potential ethical challenges and issues that may arise from the use of different types of AI in a clinical context. For example, in the context of bias, clinical decision support systems can be subject to bias arising from the unintended transfer of existing bias on the part of the developers [31,32]. Focusing on applications based on ML as part of statistical AI per definition, there is an imminent risk for bias originating from unrepresentative data sets used in the training process of the applications [33]. This highlights the importance of clearly defining and distinguishing between the various types of AI (eg, statistical or symbolic AI) to effectively address these ethical issues.

Although the integration and teaching of AI as part of medical education have been of increased scientific interest in recent years, further highlighting the need for early and adequate education of medical students, the available research is still limited [6,8,19,34]. The comparability and practical implications of current research are further limited not only due to a lack of standardization in terms of the definition of AI and possible teaching content but rather due to differences in the structure of medical education between different countries in general [19]. In Germany for example, there has been an increasing effort to define and implement AI-related competencies and learning objectives as part of medical education [35]. The recommended AI-related learning objectives are well aligned with the results of this study. Especially, the need for basic knowledge about AI models and the importance of an understanding of the data basis as well as the practical application can be confirmed by our findings [35]. But due to the lack of a uniform definition of AI within the scientific literature, the experts’ statements regarding AI models and the recommended teaching content as well as associated competencies varied in this study. Agreement on the terminology of AI and the related teaching content is especially important, as medical education should aim to provide a comparable level of knowledge and competencies for all students.

The results of this study highlight the need for comparability, as the experts’ statements not only confirm the results of current literature but further specify and highlight the importance of awareness of associated risks, critical questioning of the results, as well as the significance of basic technical and technology skills [20,25,36]. Furthermore, the results presented highlight the importance of medical education to create trust for AI-based applications, which is associated with the acceptance of the technology by its users. The highlighted significance of trust as a requirement for acceptance and the importance of being able to interpret the results is also a distinguishing feature in comparison with other publications [5,8]. Because of the significance of trust in AI on the part of the users, the need for standardization in defining and teaching AI within medical education becomes imminent, as inconsistency can lead to uncertainty and potential disapproval of the technology.

### Limitations

There are several limitations of this study. Using qualitative research methods, the level of generalization is limited due to a small sample size. Although we sought an interdisciplinary approach to the data collection, the results of the study still represent the subjective opinions of the participants. Furthermore, the results are likely to be subject to a selection bias, as no randomization was used and participants were recruited through recommendation. As only a limited number of standardized questions within the data collection were used, interviewer’s bias is also possible. Additionally, as the data collection was conducted through a web-based service provider, technical difficulties may have affected the quality of the collected data.

### Conclusions

This study aimed to explore and define relevant knowledge and understanding concerning the subject of AI in medicine as part of the medical curriculum. The results of the study, based on qualitative content analysis of expert interviews, indicate that knowledge and understanding of the fundamentals of AI, statistics, ethics, and privacy and regulation should be part of medical education. Furthermore, medical students need to be able to interpret and critically reflect on the results provided by AI, considering the associated risks and data basis. The development of trust in AI as well as the acquisition of related practical skills, including the need for basic technological skills, should be an indispensable part of medical education.

As AI in medicine is likely to become increasingly significant in the future, medical users will need adequate knowledge and understanding to use it effectively. Due to the new opportunities and challenges associated with the use of AI-based applications in medicine, medical education needs to adapt to those changes, to provide future generations of physicians with the necessary knowledge and competencies. The research aims to emphasize the importance of integrating teaching content related to AI into the medical curriculum. The results provide implications for the creation of new teaching content based on interdisciplinary data collection. Furthermore, the results further imply a need for standardization in the definition of AI as a foundation for associated teaching content and the integration of AI into medical education. Subsequent research should explore the practical implications of this study and how the results can be transferred into the medical curriculum. Furthermore, research and the development of tools are needed to assess the current knowledge and competencies of medical students regarding the use of AI in medicine. This will not only have practical implications for the creation of new teaching content but will rather allow an assessment of the success of new teaching content in the future.

## Abbreviations

AI: artificial intelligence
ML: machine learning
RCSEQ: Research Committee for Scientific Ethical Questions
